# The E3 ubiquitin ligases regulate PD-1/PD-L1 protein levels in tumor microenvironment to improve immunotherapy

**DOI:** 10.3389/fimmu.2023.1123244

**Published:** 2023-01-17

**Authors:** Bo Hou, Ting Chen, He Zhang, Jiatong Li, Peter Wang, Guanning Shang

**Affiliations:** ^1^ Department of Orthopedics, Shengjing Hospital of China Medical University, Shenyang, Liaoning, China; ^2^ Department of Biochemistry and Molecular Biology, School of Laboratory Medicine, Bengbu Medical College, Bengbu, Anhui, China

**Keywords:** E3 ligases, cancer, therapy, PD-1, PD-L1, TME

## Abstract

The tumor microenvironment (TME) is the tumor surrounding environment, which is critical for tumor development and progression. TME is also involved in clinical intervention and treatment outcomes. Modulation of TME is useful for improving therapy strategies. PD-L1 protein on tumor cells interacts with PD-1 protein on T cells, contributing to T cell dysfunction and exhaustion, blockage of the immune response. Evidence has demonstrated that the expression of PD-1/PD-L1 is associated with clinical response to anti-PD-1/PD-L1 therapy in cancer patients. It is important to discuss the regulatory machinery how PD-1/PD-L1 protein is finely regulated in tumor cells. In recent years, studies have demonstrated that PD-1/PD-L1 expression was governed by various E3 ubiquitin ligases in TME, contributing to resistance of anti-PD-1/PD-L1 therapy in human cancers. In this review, we will discuss the role and molecular mechanisms of E3 ligases-mediated regulation of PD-1 and PD-L1 in TME. Moreover, we will describe how E3 ligases-involved PD-1/PD-L1 regulation alters anti-PD-1/PD-L1 efficacy. Altogether, targeting E3 ubiquitin ligases to control the PD-1/PD-L1 protein levels could be a potential strategy to potentiate immunotherapeutic effects in cancer patients.

## Introduction

The tumor microenvironment (TME) is the tumor surrounding environment, including fibroblasts, blood vessels, different immune cells, the extracellular matrix, etc (1, 2). TME connects tumor cells and local normal cells to make them interactions, which is critical for tumor development and progression (3, 4). Moreover, tumor cells and TME influence each other. Because the TME contains some various immune cells, TME is involved in immunotherapy (5–7). TME often exhibits immunosuppressive in cancer patients, and this situation makes tumor cells evade immunologic surveillance (8). In addition, TME is critically involved in clinical intervention and changes treatment outcomes. Modulation of TME is useful for fine-tuning of therapy strategies (9–11).

The post-translational modifications (PTMs) have various types, such as acetylation, ubiquitination, phosphorylation, methylation, SUMOylation, glycosylation, and palmitoylation (12–14). The ubiquitin proteasome system (UPS) is one of PTMs to regulate protein ubiquitination and degradation (15). In general, UPS have several critical elements, such as ubiquitin, ubiquitin-activating enzyme (E1), ubiquitin-conjugating enzyme (E2), ubiquitin-protein enzyme (E3), 26S proteasome, and deubiquitinating enzymes (DUBs). E1, E2 and E3 tightly regulate the protein ubiquitination and degradation (16). E3 ligases specifically target substrates and have several classifications based on their structures, such as HECT E3 ligases, RBP E3 ligases and RING E3 ligases (17).

PD-L1 protein on tumor cells interacts with PD-1 protein on T cells, contributing to T cell dysfunction and exhaustion, including the suppression of T lymphocyte proliferation, reduction of cytokine production, blockage of the immune response (18, 19). Indeed, dysfunctional T cells have high expression of PD-1 in TME. Blockade of PD-1/PD-L1 signaling invigorates active T cells and increases immunotherapy efficacy (20, 21). Several antibodies against PD-1, such as cemiplimab (Libtayo), nivolumab (Opdivo), pembrolizumab (Keytruda), and antibodies against PD-L1, including avelumab (Bavencio), durvalumab (Imfinzi), Atezolizumab (Tecentriq), have been used in clinical trials (22–24). However, in clinical set, only a portion of patients with PD-1/PD-L1 positive tumors display a good response to anti-PD-1/PD-L1 immunotherapy (25). The poor response and adaptive immune resistance can hinder the treatment efficacy. One reason is the dynamic expression of PD-1/PD-L1 in cells because PD-1/PD-L1 expression can be induced by cytokines and multiple factors and be regulated by E3 ligases in TME (26–28). In-depth evaluation is necessary to discover how E3 ligases regulate PD-1/PD-L1 expression in TME to affect immunotherapy outcomes.

## E3 ubiquitin ligases regulate PD-1/PD-L1

Evidence has demonstrated that the expression of PD-1/PD-L1 is linked to clinical response to anti-PD-1/PD-L1 therapy in cancer patients. It is important to discover the regulatory machinery how PD-1/PD-L1 protein is finely regulated in tumor cells. In recent years, several studies have demonstrated that PD-1/PD-L1 expression was regulated by various E3 ubiquitin ligases in TME, contributing to resistance of anti-PD-1/PD-L1 therapy in human cancers (29, 30). In the following section, we will discuss the role and molecular mechanisms of E3 ligases-mediated regulation of PD-1 and PD-L1 in TME. Moreover, we will describe how E3 ligases-involved PD-1/PD-L1 regulation alters anti-PD-1/PD-L1 efficacy.

## F-box proteins

F-box protein is a subunit of SCF E3 ligase complexes and has been characterized to involve in numerous biological functions in human cancer, such as apoptosis, invasion, cell cycle, proliferation, autophagy, drug resistance, EMT, cancer stem cells and metastasis (31–34). SCF E3 ligase consists of adaptor SKP1, scaffold Cullin-1, RBX1 or RBX2 and F-box protein. So far, there are 69 F-box protein in human genome, including 37 FBXO proteins, 10 FBXW proteins, and 22 FBXL proteins (35–37). F-box proteins have been shown to regulate oncogenesis and tumor progression in numerous types of human cancers (38, 39). Liu et al. reported that FBW7-mediated PD-1 protein degradation enhanced anti-PD-1 immunotherapy in non-small cell lung cancer (NSCLC) (40). FBW7 protein is one component of the SCF E3 ubiquitin ligase, and plays a tumor suppressive role in tumorigenesis (41). FBW7 was reported to suppress M2 macrophage polarization and restrict tumor progression *via* regulation of c-Myc degradation in Lewis lung carcinoma cells (LLCs) (42). FBW7 was also identified as a new E3 ligase for PD-1 protein *via* enhancement of the K48-linked polyubiquitination of PD-1 at Lys233 site and degradation in NSCLC cells (40). CDK-1-mediated the phosphorylation of Ser261 is necessary for FBW7-involved ubiquitination of PD-1 protein. *In vivo* study used a natural terpenoids oridonin to activate FBW7 activation (43) and anti-mouse PD-1 monoclonal antibody to treat C57BL/6 mice with LLC cell injection. Combination treatment exhibited more profound tumor suppressive outcomes, which was accompanied with increased apoptosis of tumor cells and increased CD8+ CTLs infiltration. In human NSCLC tissues, FBW7 was negatively associated with PD-1 expression. Overexpression of FBW7 led to PD-1 destruction and in return promoted the blockade of PD-1/PD-L1 evasion pathway (40). High expression of FBW7 in the TME contributed to sensitivity of anti-PD-L1 therapy in NSCLC.

Another study revealed that inactivation of FBW7 effected double-stranded RNA (dsRNA) sensor expression and led to immunotherapy resistance (44). Melanoma patients had heterogeneous reactions to PD-1 blockade therapy. The resistant tumors displayed FBW7 mutations and sensitive tumors did not have the mutations of FBW7. Depletion of FBW7 in murine cancer cells resulted in resistance to PD-1 blockade in mice. Depletion of FBW7 altered TME, downregulated the expression of MDA5 and RIG1, two dsRNA sensors, and reduced the expression of MHC-1 and type I IFN (44). On the contrary, in FBW7-deficient cells, restoring MDA5 and RIG1 sensitized anti-PD-1 therapy. This work indicated that inactivation of FBW7 could be a key driver for anti-PD-1 resistance. Therefore, restoration of FBW7 might improve clinical therapeutic efficacy to anti-PD-1/PD-L1 treatment (44). FBXO38 mediated PD-1 poly-ubiquitination in K48-linked manner and proteasome degradation. In activated T cells, the PD-1 exhibited internalization and degradation (45). T cells with conditional knockout of FBXO38 promoted mouse tumor progression due to upregulation of PD-1 in tumor infiltrating T cells. Moreover, anti-PD-1 treatment abolished the efficacy of FBXO38 depletion on mouse tumor growth. The transcriptional levels of FBXO38 were decreased in tumor infiltrating T cells in mice and human cancer tissues (45). Furthermore, IL-2 treatment restored Fbxo38 transcription and promoted PD-l degradation and reduced PD-1 protein levels in T cells in mice (45). Hence, targeting FBXO38 could be a good choice to reduce PD-1 level and influence immunotherapy.

In addition, β-TrCP E3 ligase interacted with GSK3β and PD-L1, leading to the phosphorylation-dependent degradation of PD-L1 by β-TrCP. However, PD-L1 glycosylation sites at N192, N200 and N219 blocked the GSK3β binding. Moreover, EGF inactivated GSK3β and stabilized PD-L1 in breast cancer cells (46). Gefitinib suppressed EGF pathway and destabilized PD-L1, resulting in promotion of antitumor T-cell immunity and enhancement of treatment efficacy of anti-PD-1 therapy in mice. Hence, ubiquitination and glycosylation of PD-1 were involved in β-TrCP-mediated degradation of PD-1 and tumor immunotherapy efficacy (46). FBXO22 E3 ligase targeted PD-L1 for ubiquitination and degradation and increased sensitization of tumor cells to DNA damage (47). Inhibition of CDK5 elevated the expression of FBXO22 and subsequently inhibited PD-L1 protein levels, indicating that CDK5 was an upstream regulator of FBXO22 and that CDK5 inhibitors could increase the efficacy of immunotherapy in cancer cells (47).

## NEDD4 E3 ligase

FGFR3 destabilized PD-L1 *via* NEDD4 to govern T-cell-involved immune surveillance in bladder cancer (48). FGFR3, a tyrosine kinase, has been known to be overexpressed and activated in human cancers (48–50). FGFR3 alterations play an essential role in the immunotherapy for bladder cancer, including amplifications, fusions, and mutations (51). One study showed that suppression of FGFR3 increased the expression of PD-L1 level *via* modulating its ubiquitination in bladder cancer cells, contributing to blockade of anticancer activity of CD8+ T cells (48). FGFR3 had an inverse association with PD-L1 in human cancer tissues (48). NEDD4 is a HECT domain family E3 ubiquitin ligase and targets multiple substrates, including ENaC, Notch, Deltex, VEGFR2, HER3, PTEN, AMPA receptor and IGF-1R, for ubiquitination-mediated degradation, leading to regulation of cellular processes (52, 53). NEDD4 can be phosphorylated by FGFR3 and subsequently regulates PD-L1 ubiquitination in K48-linked manner. Mice with NEDD4 knockout bladder cancer displayed impaired CD8+ T cell infiltration and reduced anticancer activity because of upregulation of PD-L1. Therefore, NEDD4 E3 ligase is associated with FGFR3 targeted therapy and PD-L1 immunotherapy. Combination treatment strategy for FGFR3 and NEDD4 could be useful for bladder cancer (48).

## c-Cbl E3 ligase

It has been known that c-Cbl often acts as a tumor suppressor gene in oncogenesis (54). In immune cells, c-Cbl expression is highly expressed. One study showed that c-Cbl targeted PD-1 for proteasomal degradation and reduced PD-1 level as well as changed TME in colorectal tumors (55). In addition, c-Cbl+/- mice showed increased colorectal tumor growth and more infiltrating immune cells compared to c-Cbl wild-type mice. c-Cbl+/- mice displayed an elevated PD-1 levels in macrophages and CD8+ T lymphocytes (55). Moreover, the tumor phagocytosis in macrophages was reduced in c-Cbl+/- mice; however, this phenotype can be recovered by anti-PD-1 antibody treatment. Mechanistically, the cytoplasmic tail of PD-1 binds to C-terminus of c-Cbl, and causes c-Cbl-mediated degradation of PD-1. Hence, c-Cbl targets PD-1 expression level and alters TME, which could improve immunotherapy (55).

## SPOP E3 ligase

SPOP has been reported to participate in tumor development and progression *via* regulating its multiple substrates, including Cyclin E1, ERG, BRD4, Cdc20, TRIM24, HDAC6, Gli2, and SIRT2 (56, 57). One elegant study revealed that SPOP destructed PD-L1 protein and controlled cancer immune surveillance (58). Zhang et al. reported that PD-L1 abundance was governed by cyclin D-CDK4 and Cullin 3/SPOP. Suppression of CDK4/6 reduced phosphorylation of SPOP, and subsequently promoted APC/C Cdh1-mediated the degradation of SPOP, contributing to high expression of PD-L1 levels (58). Strikingly, loss-of-function mutations in SPOP elevated PD-L1 protein level due to dysregulation of PD-L1 degradation, conferring to reduction of TILs in human prostate cancer tissues and mouse tumor samples (58). Inhibition of CDK4/6 by inhibitors increased the anti-PD-1 immunotherapy efficacy and prolonged overall survival rates and promoted tumor regression in mice (58). Meng et al. demonstrated that Rho-associated protein kinase (ROCK) can lead to moesin phosphorylation and competing SPOP for interacting with PD-L1 (59). Blockade of ROCK by Y-27632 inhibitor or depletion of moesin reduced the expression of PD-L1, contributing to activation of T cells. Y-27632 inhibitor retarded tumor progression and promoted CD8+ and CD4+ T cell infiltration in mice *via* upregulation of multiple immune response genes (59).

Aldehyde dehydrogenase 2 (ALDH2) blocked SPOP-mediated degradation of PD-L1 *via* binding with the intracellular segment of PD-L1 (60). Silencing of ALDH2 decreased PD-L1 protein and enhanced TILs infiltration in colorectal cancer cells. Suppression of ALDH2 also caused promotion of anti-PD-1 therapeutic efficacy in colorectal cancer mouse model, indicating that ALDH2 facilitates tumor progression and enhanced immune escape *via* regulation of SPOP-mediated degradation of PD-L1 (60). Casein kinase 2 (CK2) was reported to phosphorylate PD-L1 at Thr285 and Thr290 and subsequently stabilize the PD-L1 in tumor and dendritic cells. The interaction between PD-L1 and SPOP was blocked by PD-L1 phosphorylation, contributing to protection of PD-L1 degradation. Suppression of CK2 reduced PD-L1 accumulation and increased CD80 release from dendritic cells to reactivate functions of T cells (61).

ATR inhibitor destabilized PD-L1 protein due to activation of CDK1/SPOP axis. ATR inhibitors plus anti-PD-L1 treatment led to increased innate immune activation in mice (62). Sorting nexin 6 (SNX6) can bind with Cullin 3, leading to reduction of interaction between SPOP and Cullin 3, which reduces the PD-L1 degradation. Consistently, depletion of SNX6 reduced PD-L1 protein in cancer cells (63). One research revealed that c-Myb enhanced tumor immune escape *via* targeting miR-145-5p/SPOP/PD-L1 pathway in esophageal adenocarcinoma cells (64). Specifically, c-Myb increased miR-145-5p expression and in turn reduced SPOP and regulated PD-L1, leading to suppression of T cell functions and induction of immune escape in esophageal adenocarcinoma cells (64). Cancer stem cell-derived exosomal miR-17-5p reduced SPOP expression and increased PD-L1 accumulation, leading to suppression of antitumor immunity in colorectal cancer cells (65). Recently, SPOP was found to increase the movement of PD-1 away from PD-L1 in spatial localization, and enhanced tumor metastasis in cervical cancer (66). In ovarian cancer cells, Cullin 3/SPOP facilitated sensitivity of chemotherapy and blocked immune escape *via* promoting PD-L1 protein degradation (67). Recently, SPOP mutations were revealed to enhance tumor immune escape through targeting the interferin regulatory factor 1 (IRF1)-PD-L1 axis in endometrial cancer (68). Taken together, SPOP E3 ligase targets PD-L1 degradation and involves in anti-PD-1 immunotherapy in human cancers.

## Other E3 ligases

Inflammation-related molecule A20 (also known as TNFAIP3) plays an essential role in antitumor immunity and inflammation *via* negative regulation of NF-κB pathway (69). Melanoma patients with upregulation of A20 displayed poor treatment effect to anti-PD-1 therapy and reduced CD8+ T cell activity. Modulation of A20 regulated PD-L1 expression and invigorated CD8+ T cell infiltration, leading to enhancement of immunotherapy (70). A20, acting as an E3 ubiquitin, ligase, activated STAT3 ad PD-L1 expression due to promotion of prohibitin ubiquitination and degradation (70). CDK5 inhibited the PPARγ E3 ligase activity and protected ESRP1 from degradation. CDK5 triggered Ser 273 phosphorylation of PPARγ and switched CD44 isoform from CD44s to CD44v, leading to promoting TNBC CSCs development (71). Inactivation of CDK5 and phosphorylation of PPARγ suppressed the numbers of CD44v+ breast CSCs in tissues, which inhibited tumor metastasis in TNBC mice. Blockade of stemness transformation facilitated anti-PD-1 treatment outcomes *via* reversing tumor immunosuppressive microenvironment in TNBC (71). KLHL22 is an adaptor of Cullin 3 E3 ligase and mediated the ubiquitination and degradation of PD-1. Therefore, deficiency of KLHL22 resulted in PD-1 upregulation, conferring to suppression of T cells-mediated antitumor response and facilitated tumor development (72). In clinical colorectal cancer patients, there was a downregulation of KLHL22 in tumor infiltrating T cells. 5-fluorouracil (5-FU) could suppress the KLHL22 transcription and elevate the expression of PD-1 (72). Hence, KLHL22 governed excessive T cell suppression *via* regulation of PD-1 expression in colorectal cancer. STUB1 was reported to act as an E3 ligase and lead to destabilization of PD-L1. A type-3 transmembrane protein CMTM6 could maintain PD-L1 *via* blocking ubiquitination in tumor cells (73). CMTM4 displayed the similar function in regulation of PD-L1 protein levels. CMTM6 accelerated the ability of tumor cells with PD-L1 expression to repress T cells (73). TMUB1 was identified as a modulator of PD-L1 PTMs, which competed with HUWE1 to bind with PD-L1 and suppressed its ubiquitination at K281 in the ER. TMUB1 recruited STT3A and accelerated PD-L1 N-glycosylation and stability, resulting in enhancement of PD-L1 maturation and contribution of immune evasion. A peptide that competed with TMUB1 elevated anticancer immunity and retarded tumor growth in mice (74).

## Deubiquitinases stabilize PD-1/PD-L1

DUBs can cleave and remove ubiquitins from molecules, which is classified into two groups: metalloproteases and cysteine proteases. DUBs stabilize the protein levels of PD-1 and PD-L1 in cancer cells. For instance, COP9 signalosome 5 (CSN5) maintained PD-L1 protein accumulation *via* inhibition of ubiquitination and degradation of PD-L1 (75). TNF-α induced PD-L1 stabilization due to p65-mediated induction of CSN5 in cancer cells. Suppression of CSN5 by natural agent curcumin sensitized tumor cells to anti-CTLA4 blockade because of diminishing PD-L1 expression (75). The deubiquitinase USP22 can bind with PD-L1 and enhance its stability, leading to reduced T cell cytotoxicity in tumor cells (76). Similarly, another group also identified that USP22 can target PD-L1 and resulted in suppression of antitumor immunity (77). USP7 depletion led to downregulation of PD-L1 and caused sensitization of T cells killing in gastric tumor cells (78).

USP8 depletion enhanced immunotherapy by regulation of TME *via* targeting PD-L1 ubiquitination and activating the infiltrated CD8+ T cells (79). In line with this report, USP8 deubiquitinated PD-L1 and upregulated its expression in pancreatic cancer. Anti-PD-L1 in combination with a USP8 inhibitor attenuated tumor growth *via* activation of cytotoxic T cells (80). OTUB1 blocked ER-associated degradation of PD-L1 and triggered immunosuppression in tumor cells (81). Interestingly, depletion of USP12 established a tumor-promoting TME due to insufficient deubiquitination of PPM1B and activation of NF-κB in cancer cells, which contributed to desensitization of anti-PD-1 immunotherapy in lung cancer cells (82). In addition, USP14 stabilized indoleamine 2,3 dioxygenase 1 (IDO1) and enhanced immune suppression in colorectal cancer (83). Depletion of USP14 promoted anti-PD-1 responsiveness in mice and reversed inhibition of cytotoxic T cells due to inhibition of IDOI (83).

## Compounds target E3 ligases to regulate PD-1/PD-L1

Evidence has revealed that several compounds regulate the expression of E3 ubiquitin ligases and modulate the expression of PD-1/PD-L1 and change the immunotherapy efficacy in human cancers. For example, 2,5-dimethylcelecoxib (DMC) induced hepatitis B virus X (HBx)-mediated PD-L1 ubiquitination and improved TIME in HCC (84). DMC, an inhibitor of mPGES-1, has been reported to repress HBV-involved HCC progression. DMC elevated the CD8+ T cell infiltrations in HCC mouse model, and mouse tumor tissues displayed the downregulation of PD-L1 and CD163. The combination of DMC and atezolizumab exhibited more significant anticancer efficacy. DMC enhanced RBX1 E3 ligase-mediated PD-1 degradation *via* activation of AMPK pathway in HCC cells (84). Avadomide is cereblon E3 ligase modulator and upregulates the expression of PD-L1 in CLL cells (85). Avadomide activated interferon (IFN) signaling in T cells and reactivated T cell responses and promoted chemokine expression. PDX mice displayed CD8+ T cell-inflamed TME after avadomide treatment and increased the sensitivity of anti-PD-1/PD-L1 therapy (85). Hence, avadomide could enhance sensitivity of immunotherapy in CLL cells *via* targeting IFN signaling pathway.

APG-115, an inhibitor of MDM2 E3 ligase, synergized with anti-PD-1 therapy *via* promoting anticancer immunity in the TME (86). It has been shown that p53 activation inhibited M2 macrophage polarization in myeloid. APG-115 treatment increased activation of p53 and p1 in bone marrow-derived macrophages and decreased c-Myc and c-Maf and caused a reduction number of immunosuppressive M2 macrophage (86). Mice with APG-115 treatment elevated M1 macrophage polarization in the spleen. Moreover, APG-115 in combination with anti-PD-1 therapy contributed to enhanced tumor suppressive activity in mice (86). MET inhibitors, capmatinib and tivantinib, enhanced tumor evasion of the immune response by stabilization of PD-L1 in HCC (87). MET inhibitors elevated PD-L1 expression and blocked the antitumor ability of T cells. Mechanistically, suppression of MET blocked GSK3β phosphorylation and promoted the interaction between TRAF6 and GSK3β, leading to inactivation of GSK3β because of TRAF6-induced GSK3β K63 ubiquitination, which facilitated the PD-L1 expression in HCC cells (87). Metformin attenuated the stability and membrane localization of PD-L1 and elevated the activity of CTL. Metformin activated AMPK and phosphorylated PD-L1 at S195 site, resulting in abnormal PD-L1 glycosylation and its ER accumulation and ERAD (88).

## PROTACs target PD-1/PD-L1 for improving immunotherapy

PROTACs is a new technology for regulating a protein of interest (POI) by degradation by specific E3 ligases (89, 90). Numerous of E3 ligases, including cereblon, MDM2, β-TrCP, and VHL, have been applied in PROTAC strategy (91). PROTAC is a ternary complex that links a POI ligand to an E3 ligase *via* an optimal linker (92, 93). PROTACs have been reported to target several important signaling pathways in TME and improve antitumor therapy (94). Peptide-based PROTAC of FOXM1 inhibited the expression of PD-L1 and glucose transporter GLUT1 and attenuated carcinogenesis (95). FOXM1-PROTAC mediated degradation of FOXM1 protein in tumor cells and suppressed viability, migration and invasion in several types of tumor cells. In HepG2 and MDA-MB-231 tumor cell xenograft mice, FOXM1-PROTAC retarded tumor growth (95). Moreover, FOXM1-PROTAC reduced the expression of PD-L1, indicating that PROTACs might be used for targeting PD-L1 degradation to improve the immunotherapy in human cancer.

One group developed a new PROTAC molecule 21a that enhanced PD-L1 protein degradation in several types of tumor cells, suggesting that compound 21a might be an alternative way for immunotherapy in cancer patients (96). Another group designed a new resorcinol diphenyl ether-based PROTAC, compound P22, that impaired the interaction between PD-1 and PD-L1 and reactivated the immunity. P22 compound decreased the protein levels of PD-L1 *via* lysosome-mediated degradation and affected immune functions (97). Degradation of BET protein by the PROTAC technology induced death receptor 5 (DR5)-involved immunogenic cell death (ICD) in colorectal cancer cells, leading to colorectal cancer progression and enhancement of anti-PD-1 antibody blockade (98). Targeting SHP2, a protein tyrosine phosphatase, by PROTACs is useful for cancer immunotherapy partly *via* regulation of several signaling pathways, such as PD-1/PD-L1, PI3K/AKT, RAS/ERK, JAK/STAT pathways (99). Moreover, nano-PROTACs targeting SHP2 inactivated the CD47/SIRPα and PD-1/PD-L1 pathways, contributing to reinvigoration of T cells and macrophages as well as promotion of antitumor immune response (100). Recently, peptide-PROTACs targeting PD-1/PD-L1 degradation were designed and exhibited high potential activity to degrade PD-1/PD-L1 in tumor cells, which caused tumor cell death (101). PROTACs targeting hematopoietic progenitor kinase1 (HPK1) regulated T cell function and potentiated the efficacy of CAR-T cell-based immunotherapies (102). Cotton et al. developed antibody-based PROTACs (AbTACs) to disrupt PD-L1 protein stability. AbTACs recruited RNF43 E3 ligase and induced the lysosomal degradation of PD-L1 (103). These reports decipher that PROTACs are novel compounds for targeting PD-1/PD-L1 or other critical factors in immunosuppressive pathways to improve the efficacy of immunotherapy.

## Discussion and perspectives

**Figure 1:**
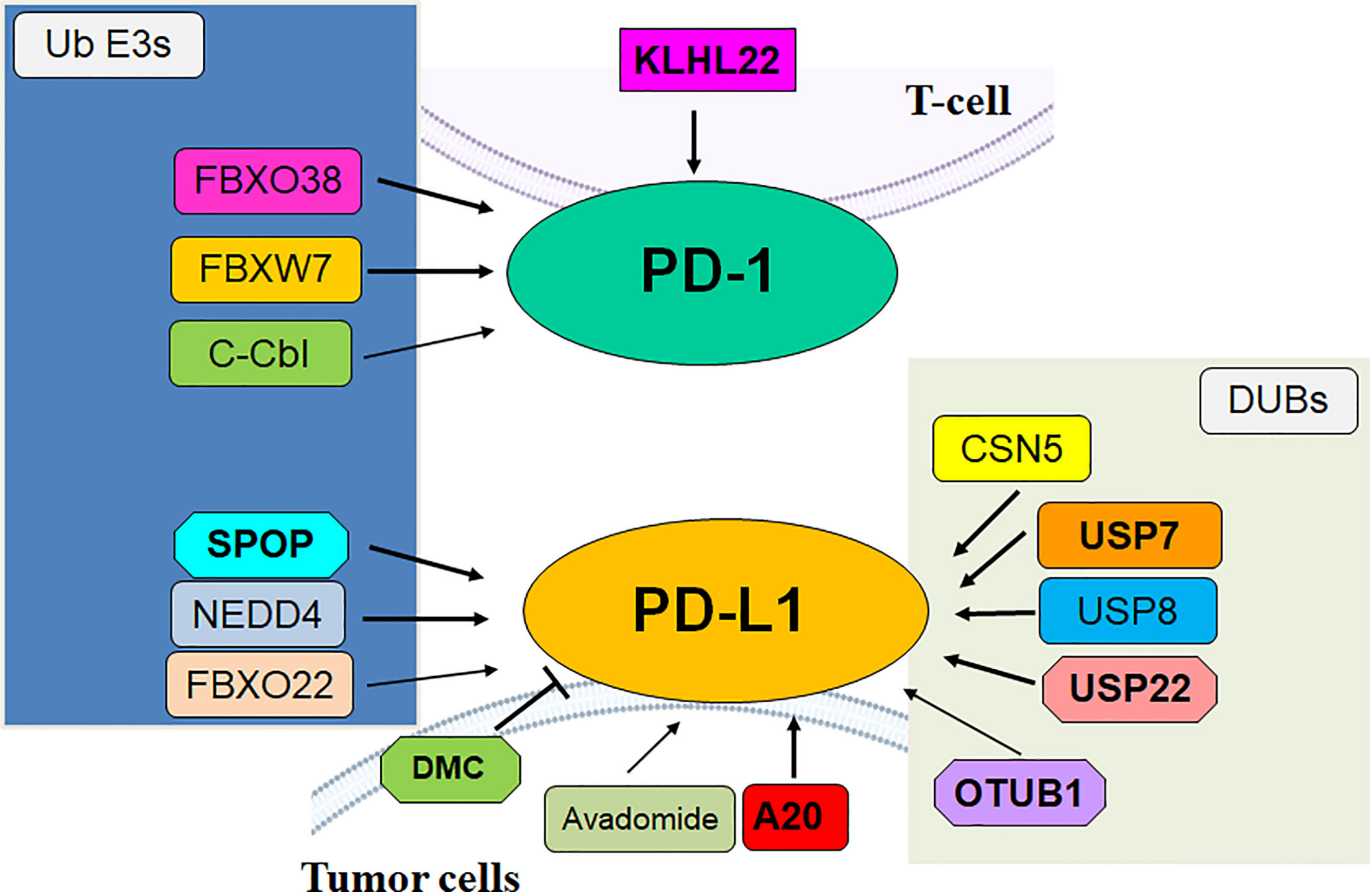
The role of E3 ubiquitin ligases and DUBs in regulation of PD-1/PD-L1 in cancer.

In conclusion, the E3 ligases govern the ubiquitination and degradation of PD-1/PD-L1 in the TME (**Table 1**). Targeting E3 ligases might be a potential strategy for promoting antitumor immunity in human cancer (**Figure 1**). Several Strategies have been proposed for developing PD-1 inhibitors, including PROTACs (104). It is important to note several issues for readers to fully understand the role of E3 ubiquitin ligases to regulate TME and PD-1/PD-L1 in immunotherapy. Some E3 ubiquitin ligases regulated specific substrates, not PD-1/PD-L1 proteins, to influence TME in tumor cells. FBXL8 downregulation increased accumulation of CCND2 and IRF5 and reduced the cancer-promoting chemokines, modulated TME, leading to repressing tumor metastasis in breast cancer (105). COP1 E3 ligase knockdown reduced chemokine secretion and macrophage infiltration, increased immune checkpoint blockade efficacy, promoted tumor suppressive immunity in the TME. COP1 acted as the E3 ligase to induce polyubiquitination and degradation of the C/ebpδ, resulting in activation of macrophage chemoattractant genes (106).

**Table 1:** The E3 ubiquitin ligases regulate PD-1/PD-L1 protein levels.

Items	Targets	TME	Functions	Ref.
FBXW7	Targets the K48-linked polyubiquitination of PD-1 at Lys233 site and degradation.	Increases apoptosis of tumor cells and increased CD8+ CTLs infiltration.	Enhances anti-PD-1 immunotherapy.	(40)
FBXO38	Targets PD-1 poly-ubiquitination in K48-linked manner and proteasome degradation.	IL-2 restores Fbxo38 transcription and promotes PD-l degradation in tumor infiltrating T cells.	Anti-PD-1 treatment abolishes the efficacy of FBXO38 depletion on mouse tumor growth.	(45)
FBXO22	Targets PD-L1 for ubiquitination and degradation.	CDK5 was an upstream regulator of FBXO22 and CDK5 inhibitors increase the efficacy of immunotherapy.	Increased sensitization of tumor cells to DNA damage.	(47)
NEDD4	NEDD4 is phosphorylated by FGFR3 and regulates PD-L1 ubiquitination in K48-linked manner.	Mice with NEDD4 knockout bladder cancer display impaired CD8+ T cell infiltration and reduces anticancer activity.	NEDD4 E3 ligase is associated with FGFR3 targeted therapy and PD-L1 immunotherapy.	(48)
c-Cbl	The cytoplasmic tail of PD-1 binds to C-terminus of c-Cbl, leading to degradation of PD-1.	c-Cbl targets PD-1 expression level and alters TME.	c-Cbl improves immunotherapy.	(55)
SPOP	PD-L1 abundance is governed by cyclin D-CDK4 and Cullin 3/SPOP.	Loss-of-function mutations in SPOP elevated PD-L1 protein level, conferring to reduction of TILs in human prostate cancer tissues.	Inhibition of CDK4/6 by inhibitors increased the anti-PD-1 immunotherapy efficacy.	(58)
A20	Modulation of A20 regulated PD-L1 expression.	Modulation of A20 invigorated CD8+ T cell infiltration.	Modulation of A20 enhances immunotherapy.	(70)
KLHL22	Deficiency of KLHL22 results in PD-1 upregulation.	Deletion of KLHL22 leads to suppression of T cells-mediated antitumor response.	Deficiency of KLHL22 facilitated tumor development.	(72)

Growth differentiation factor 15 (GDF15) facilitated tumor immunosuppression *via* interaction with CD48 on Treg cells and downregulation of E3 ligase STUB1, leading to accumulation of FOXP3 protein in liver cancer (107). UBR5 E3 ligase augmented immunosuppressive macrophage and modulated the TME and facilitated tumor growth and metastasis in ovarian cancer *via* maintaining β-catenin signaling (108). NEDD4 E3 ligase suppressed T-cell-induced antitumor immunity *via* targeting the immune checkpoint GITR for degradation in melanoma (109). Depletion of RNF43 E3 ligase remodeled the TIME and facilitated Kras-induced oncogenesis in pancreatic cancer (110). TRAF6 E3 ligase reshaped TME by increasing the immunosuppressive functions of MDSCs *via* targeting K63-linked ubiquitination and phosphorylation of STAT3 (111). Knockdown of E3 ubiquitin ligase Cbl-b increased CAR T-cell effects and blocked CD8+ T-cell exhaustion, upregulated the expression of IFN-γ and TNFα, and accelerated tumor cell killing (112). Calponin 1 can bind with PDLIM7 and protect its disruption by the NEDD4-1, resulting in activation of ROCK1/MLC pathway. Calponin 1 elevated matrix stiffness in CAF and enhance 5-Fu chemoresistance by activation of YAP in gastric cancer (113).

Senescent stromal cells develop epiregulin (EREG), a member of the EGF family. EREG, a ligand of EGFR, can regulate EGFR-induced oncogenesis. EREG inhibits cellular sensitivity to TKIs treatment (114). High expression of EREG was associated with tumor stage, metastasis and survival in human cancer patients. DNA-damaging agents (DDAs), such as bleomycin, mitoxantrone, and doxorubicin, induced the expression of EREG in stromal cells (115). Stromal EREG levels were linked to adverse clinical outcomes. Moreover, EREG upregulation was due to activation of NF-κB signaling pathway. Furthermore, stromal EREG changed recipient tumor cell phenotypes in prostate cancer cells (115). MARCHF4 (membrane associated ring-CH-type finger 4), a member of E3 ubiquitin protein ligase, promoted viability of prostate cancer cells after mitoxantrone treatment. Overexpression of MARCHF4 induced EMT in prostate cancer cells. MARCHF4 overexpression caused chemotherapeutic agent resistance in prostate cancer cells. Targeting EREG in the damaged TME enhanced treatment efficacy in mice (115). Stromal cell-derived EREG-mediated drug resistance was partly due to MARCHF4 upregulation in recipient tumor cells (115).

HDM201, an inhibitor of MDM2 E3 ligase, has been reported to increase the numbers of dendritic cells in mice (116). Moreover, HDM201 elevated the CD8+/Treg ration and promoted the numbers of Tbet+Eomes+CD8+ T cells in tumor, and this phenotype alteration was abrogated by p53 depletion in tumor cells. Anti-PD-1/PD-L1 therapy in combination with HDM201 enhanced tumor regressions (116). Notably, the function of HDM201 in tumorigenesis is dependent on induction of antitumor immunity. Suppression of MDM2 by its inhibitors stimulated adaptive immunity, which can be promoted by inactivation of PD-1/PD-L1 pathway in cancer patients with p53 wild-type tumors (116). Therefore, inhibition of MDM2 E3 ligase increased anti-PD-1/PD-L1 therapeutic efficacy *via* regulation of immune and stromal microenvironment in p53 wild-type cancer patients. Trim35 E3 ligase influenced the TIME and reduced the DLBCL progression *via* targeting a regulator of circadian rhythmicity CLOCK for degradation and modulating NK cell infiltration (117). Galectin-9 augmented an immunosuppression in TME *via* accelerating TRIM29-mediated degradation of STING in human cancers (118). FBW7-induced degradation of ZEB2 was reported to associate with EMT and TME, resulting in enhancement of colorectal CSCs and drug resistance (119). Depletion of stromal hedgehog signaling Smoothened promoted proliferation of pancreatic cancer *via* initiating RNF5-induced degradation of PTEN and subsequent activation of AKT (120). In addition, UBR5 E3 ligase accelerated tumor growth and metastasis *via* regulation of apoptosis, necrosis, EMT and angiogenesis in TNBC (121). Besides, ubiquitination of PD-1/PD-L1, its phosphorylation, glycosylation, palmitoylation, and acetylation have been reported (28, 122). Most studies focused on PD-1 and PD-L1 regulations, whereas PD-L2 regulation by E3 ligases was largely unclear. It is pivotal to determine the PTM regulatory mechanism of PD-L2 in cancer immunotherapy. Taken together, targeting E3 ubiquitin ligases to modulate the PD-1/PD-L1 protein levels might be a promising approach to improve immunotherapeutic effects in cancer patients.

## Author contributions

BH and TC wrote the manuscript. HZ and JL made the table and figures. PW and GS edited and revised the manuscript. All authors contributed to the article and approved the submitted version.
